# Factors associated with Gla-rich protein serum concentrations in healthy adults

**DOI:** 10.11613/BM.2026.020709

**Published:** 2026-06-15

**Authors:** Catarina Marreiros, Carla Viegas, Selene Nunes, Ana Paula Silva, Marília Faísca, Leon Schurgers, Dina Costa Simes

**Affiliations:** 1 Centre of Marine Sciences (CCMAR/CIMAR LA), Universidade do Algarve, Faro, Portugal; 2 GenoGla Diagnostics, Centro de Ciências do Mar do Algarve (CCMAR/CIMAR LA), Universidade do Algarve, Faro, Portugal; 3 Department of Imunology, Unidade Local de Saúde do Algarve (ULSA), Faro, Portugal; 4 Department of Nephology, Unidade Local de Saúde do Algarve (ULSA), Faro, Portugal; 5 Faculty of Medicine and Biomedical Sciences, Universidade do Algarve, Faro, Portugal; 6 SYNLAB Algarve, Faro; Portugal; 7 Department of Biochemistry, Cardiovascular Research Institute Maastricht (CARIM), Maastricht University, Maastricht, The Netherlands

**Keywords:** Gla-rich protein, serum concentrations, healthy adults, age factors

## Abstract

**Introduction:**

Gla-rich protein (GRP) is a vitamin K-dependent protein involved in the regulation of ectopic calcification and inflammatory processes. However, data on circulating GRP concentrations in healthy adults remain limited.

**Materials and methods:**

This cross-sectional observational study evaluated serum total GRP (tGRP) concentrations in 254 healthy adult blood donors (48% male), aged 18-65 years, using a sandwich enzyme-linked immunosorbent assay (ELISA) (GenoGla Diagnostics, Faro, Portugal). Participants were stratified by age tertiles, and associations between serum tGRP concentrations and demographic and laboratory parameters were assessed using non-parametric statistical tests, Spearman correlation, and stepwise linear regression.

**Results:**

The median serum tGRP concentration in the overall population was 1809.5 pg/mL (interquartile range: 1197.4-2476.0 pg/mL). Serum tGRP concentrations differed significantly across age tertiles (P = 0.038), with higher concentrations observed in younger adults. Serum tGRP concentrations showed a weak but statistically significant inverse correlation with age (r = - 0.30, P = 0.001), while no significant differences were observed between sexes (P = 0.420). In multivariable analysis, age and serum calcium concentration remained independently associated with serum tGRP concentrations, jointly explaining approximately 7% of their variability.

**Conclusion:**

This study establishes robust reference data for circulating tGRP concentrations in healthy adults, providing a foundation for the interpretation of GRP measurements in future clinical and epidemiological research.

## Graphical abstract

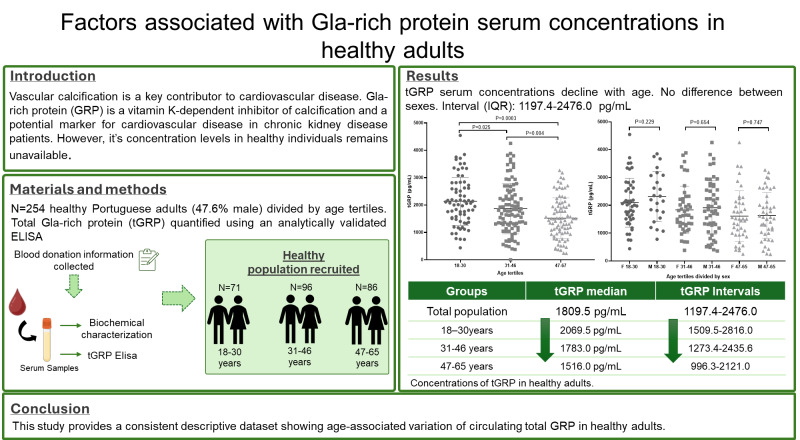

## Highlights

• Age had a weak negative association with total Gla-rich protein (tGRP)

• No significant sex differences were observed in tGRP concentrations

• Highest tGRP concentrations observed in the 18-30 age group

• Age and calcium were the main factors influencing serum tGRP variability

## Introduction

Gla-rich protein (GRP), also known as upper zone of growth plate and cartilage matrix associated protein (UCMA), is a circulating vitamin K-dependent protein with calcium- and mineral-binding properties (*1*-*3*). Gla-rich protein has been implicated in biological processes related to extracellular matrix organization mineral homeostasis, inflammatory responses, and mechanisms driving malignant transformation (*4*-*8*).

Most available knowledge regarding GRP derives from studies conducted in pathological contexts, particularly conditions characterized by inflammation and ectopic calcification (*9*-*11*). Accordingly, circulating GRP concentrations have been reported in several diseases cohorts, including chronic kidney disease and cardiovascular disease (*12*-*16*).

However, data on circulating GRP concentrations in well-characterized healthy adult populations remain limited. To date, GRP concentrations have mainly been reported in control groups, not specifically designed to represent healthy individuals and therefore provides limited insight into GRP concentrations in this context (*15*, *16*).

Reliable characterization of circulating GRP concentrations in healthy populations requires analytical robustness. Our group has previously reported GRP concentrations in several clinical cohorts using a sandwich enzyme-linked immunosorbent assay (ELISA) for total GRP (tGRP). Since its initial description this assay has undergone technical optimization, aimed at improving analytical robustness and long-term performance while preserving the original assay principle and analytical target (*17*). Importantly, all previously reported clinical GRP measurements from our group were generated using this optimized assay version; however, its formal analytical validation is reported for the first time in the present study (*12*-*14*).

Establishing descriptive data on GRP concentrations in healthy adults is essential for contextualizing findings from disease-based studies and for supporting future clinical and translational research. In healthy individuals, demographic characteristics such as age and sex, as well as routinely measured laboratory parameters reflecting renal function, lipid metabolism, and low-grade inflammation, may plausibly influence circulating protein concentrations and therefore warrant systematic evaluation.

Therefore, the aim of the present study was to describe serum tGRP concentrations in a cohort of healthy adult blood donors using a sandwich ELISA and to explore their associations with demographic characteristics and selected routine laboratory parameters.

## Materials and methods

### Subjects

This cross-sectional observational study evaluated serum tGRP concentrations in healthy adult blood donors. Participants were recruited at the Immunology Department of Algarve Local Health Unit (ULSA), Faro, Portugal, between May and August 2024. A total of 363 blood donors were selected prior to blood collection using a standardized health questionnaire and medical interview, mandatory for all blood donation units in Portugal and regulated by the Portuguese Institute of Blood and Transplantation (IPST). This screening procedure follows national legislation transposing European Union directives on blood quality and safety (Directive 2002/98/EC and subsequent implementing directives) and includes evaluation of medical history, medication use, recent infections, and transfusion-relevant risk factors. As part of routine blood donor eligibility screening, hemoglobin concentration was assessed by trained nursing staff using a point-of-care capillary finger-prick hemoglobin test (Werfen, Carnaxide, Portugal). Individuals not meeting blood donation hemoglobin requirements were excluded as viable blood donors. Only donors fulfilling all eligibility requirements for blood donation were selected for posterior biochemical analysis (Table 1).

**Table 1:** Inclusion criteria and parameters obtained for the selection of apparently healthy subjects

**Parameter (unit)**	**Inclusion criteria**	**Participant values**
Age, years	18-65*	19-65
BMI, kg/m^2^	< 30*	24.90 ± 3.14
Creatinine, µmol/L	62-115^†^	73 (64.5-86.6)
eGFR, mL/min/1.73 m^2^	≥ 90^†^	112 (108-124)
Total cholesterol, mmol/L	< 5.70^†^	4.64 (4.03-5.20)
HDL cholesterol, mmol/L	> 0.91^†^	1.45 (1.19-1.57)
LDL cholesterol, mmol/L	< 3.37^†^	2.69 (2.16-3.03)
Total protein, g/L	60-83^†^	72.50 ± 5.80
Calcium, mmol/L	2.12-2.62^†^	2.39 ±0.13
Phosphate, mmol/L	1.10-1.45^†^	1.24 ±0.08
Magnesium, mmol/L	0.70-0.90^†^	0.77 ± 0.05
Sodium, mmol/L	136-145^†^	139.2 ± 2.65
Potassium, mmol/L	3.7-5.2^†^	4.28 ± 0.39
Total bilirubin, µmol/L	< 20^†^	9.75 (3.72-12.45)
AST, U/L	< 40^†^	22.80 (17.24-26.12)
hsCRP, mg/L	≤ 3^†^	0.40 (0.26-0.45)
Characteristics and inclusion criteria. Data are represented mean ± standard deviation or median (interquartile range), according to data distribution. Age is presented as minimum-maximum. *Inclusion criteria applied during blood donor selection according to ULSA institutional blood donation eligibility requirements. ^†^Biochemical reference ranges used to exclude participants with values outside the normal laboratory range (Synlab, Portugal).^.^ BMI - body mass index. eGFR - estimated glomerular filtration rate. C-HDL - high density lipoprotein. C-LDL - low density lipoprotein. AST - aspartate aminotransferase. hsCRP - high sensitivity C-reactive protein.		

Exclusion criteria comprised obesity (body mass index ≥ 30 kg/m^2^), known pregnancy, non-Caucasian ancestry, a previous or current diagnosis of diabetes mellitus (type 1 or type 2), thyroid, liver or kidney disease, anemia, osteoarthritis or other articular disease, and cardiovascular disease (including hypertension, coronary artery disease, previous myocardial infarction, coronary angioplasty, coronary bypass surgery, varicose veins, congestive cardiac failure, or stroke). Individuals with a first-degree family history of hypertension, neoplasia, or diabetes mellitus (type 1 or type 2), and/or laboratory parameters outside predefined eligibility ranges for the present study (Table 1) were excluded. These reference ranges correspond to routine clinical chemistry parameters measured as part of the study and were used to ensure inclusion of individuals without biochemical abnormalities. In addition, participants receiving coumarin derivatives, systemic glucocorticoids or other anti-inflammatory therapy, or calcium/phosphate binders were excluded. Information on prescription medication use was systematically collected during donor eligibility assessment. The use of over-the-counter dietary supplements, such as vitamins or mineral preparations, was not systematically recorded. Body mass index (BMI) was calculated as weight divided by height squared (kg/m^2^). Estimated glomerular filtration rate (eGFR) was calculated using the CKD-EPI equation. The study was conducted in accordance with the Declaration of Helsinki. All participants provided written informed consent for the use of blood samples and associated data for research purposes, according to ULSA blood donation procedures. Ethical approval for sample collection, storage, and use in this study was obtained from the Ethics Committee of ULSA (approval code UAIF063/2021).

Venous blood was collected from the antecubital vein under resting conditions during routine blood donation procedures. Because sampling occurred throughout the day, participants were not required to fast. Blood was collected into serum tubes without anticoagulant (Greiner Bio-One, São Paulo, Brasil). Samples were allowed to clot for 30-60 minutes at room temperature and centrifuged for 10 minutes at 2000xg at room temperature. Serum samples were anonymized using a coded identification system and stored at - 80 °C at ULSA.

After completion of participant recruitment, serum samples were transported in a single batch to the Centre of Marine Sciences (CCMAR), University of Algarve, under temperature-controlled conditions on dry ice and stored at - 80 °C until analysis. All samples underwent two freeze-thaw cycles: the first for routine biochemical analysis, and the second for serum tGRP quantification.

Routine clinical chemistry parameters were measured in September 2024 using standard automated methods at SYNLAB facilities (Faro, Portugal) on an ADVIA 1800 Clinical Chemistry System (Siemens Healthineers, Erlangen, Germany). Parameters included high-sensitivity C-reactive protein (hsCRP), creatinine, calcium, phosphate, magnesium, potassium, sodium, total bilirubin, aspartate aminotransferase (AST), total protein, total cholesterol, HDL-cholesterol, and LDL-cholesterol.

### Methods

Serum tGRP concentrations were measured in December 2024 using a proprietary in-house sandwich ELISA developed by GenoGla Diagnostics (Faro, Portugal), based on the assay originally described (*17*). The assay uses an affinity-purified goat polyclonal antibody, as capture antibody, directed against the N-terminal region of GRP (N-TermGRP), designed to recognize the GRP-F1 isoform.

Minor protocol adjustments relative to the original assay were introduced while maintaining the same analytical principle (*17*).

Calibration curves were generated using serial dilutions of RP-HPLC-purified recombinant GRP-F1 standards (*18*). The validated quantitative range of the assay was 240-1600 pg/mL. Analytical performance was evaluated according to standard validation procedures.

Linearity was assessed by least-squares linear regression, with acceptance criterion defined as R^2^ ≥ 0.99.

Intra-assay precision was assessed by calculating the coefficient of variation (CV, %) for replicate measurements within a single assay run. Inter-assay precision was assessed using in-house quality control (QC) serum samples at two concentration levels (QC Low and QC High), QC samples were analyzed across independent assay runs performed within the same year. Inter-assay precision is expressed as the coefficient of variation (CV, %) for each QC level on an annual basis.

Analytical sensitivity was determined by calculating the limit of detection (LOD) as the mean blank signal plus three standard deviations. The lower limit of quantification (LLOQ) was defined as the lowest concentration meeting predefined accuracy and precision criteria.

### Statistical analysis

Statistical analysis were performed using SPSS (version 26; IBM, Chicago, IL, USA). Normality of continuous variables was assessed using the Shapiro-Wilk test. Variables with a non-Gaussian distribution are presented as median with interquartile range (IQR, 25th-75th percentiles). Variables with a Gaussian distribution are presented as mean ± standard deviation (SD). Categorical variables are presented as absolute and relative frequencies. Participants were stratified into three age groups based on tertiles of the age distribution (18-30, 31-46, and 47-65 years). For descriptive and comparative analyses, serum tGRP concentrations were also dichotomized using the population median value. Comparisons between two independent groups (sex groups) were performed using the Mann–Whitney U test. Differences across age groups were assesed using the Kruskal-Wallis test with Dunn’s *post hoc* correction for multiple comparisons. Exploratory associations of continuous variables were made using Spearman’s rank correlation coefficient, and results are reported with corresponding 95% confidence intervals. For categorical comparisons between age subgroups, the chi-square test was used, followed by *post hoc* pairwise comparisons using the z-test for proportions with Bonferroni correction. To identify independent predictors of circulating tGRP concentrations, a stepwise linear regression analysis was performed. As serum tGRP concentrations were continuously distributed but showed a non-normal distribution, logarithmic transformation was applied prior to regression analysis to approximate normality and satisfy model assumptions. Variables showing a statistically significant association with tGRP concentrations in univariable analyses (ρ < 0.05) were eligible for inclusion in the multivariable model. All statistical tests were two-sided, and a P value ≤ 0.05 was considered statistically significant.

## Results

### Analytical performance of the optimized Gla-rich protein ELISA

The analytical performance of the optimized sandwich ELISA for serum tGRP was evaluated.

The assay demonstrated good linearity over the evaluation period (2018-2025), with high goodness of fit across the measuring range (R^2^ ≥ 0.993), Table 2. Assay precision was acceptable, with intra-assay CV ranging from 1.4% to 3.2% and inter-assay CV ranging from 2.5% to 9.4%. The lower limit of quantification (LLOQ) remained stable across years (240-280 pg/mL), supporting reliable quantification of circulating tGRP.

**Table 2:** Long-term precision and analytical sensitivity parameters of the total Gla-rich protein ELISA

**Year**	**Intra-assay CV** **(%)**	**Inter-assay CV (%) - QC Low**	**Inter-assay CV (%) - QC High**	**LOD** **(pg/mL)**	**LLOQ** **(pg/mL)**
2018	2.7 (2.0-4.6)	9.1	5.9	55.8 (8.4-105.2)	240 (240-240)
2019	2.5 (2.0-3.3)	9.0	4.8	27.1(0-61.0)	280 (240-320)
2021	2.4 (2.2-5.0)	8.6	2.5	0 (0-5.1)	240 (240-320)
2022	2.0 (1.5-3.2)	7.6	5.4	47.5 (17.4-102.5)	240 (240-320)
2023	2.1 (0.7-4.2)	8.8	4.8	48.6 (10.8-132.6)	240 (240-240)
2024	1.5 (0.9-1.8)	8.8	3.4	51.1(50.3-106.6)	240 (240-240)
2025	1.5 (1.0-4.1)	9.4	5.3	15.1 (0-55.9)	240 (240-280)
Data are presented as mean ± standard deviation for intra-assay coefficient of variation (CV) and as absolute values for inter-assay CV (QC Low and QC High). LOD and LLOQ values are presented as median (minimum-maximum). QC - quality control. LOD - limit of detection. LLOQ - lower limit of detection. IQR - interquartile range.					

### Circulating total Gla-rich protein concentrations in healthy individuals

**Figure 1:**
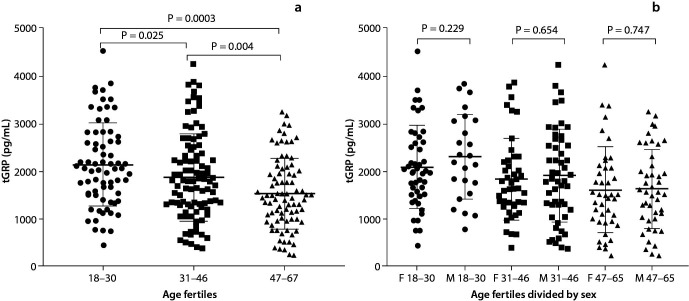
Total GRP (tGRP) serum concentrations across age groups (a) and segmented by sex (b), for the 254 individuals meeting the inclusion criteria (*i.e*., 18-30 years *vs.* 31-46 years *vs.* 47-65 years). Data are presented as individual values with median and range for tGRP serum concentrations. (a) Kruskal-Wallis with Dunn’s *post hoc* analysis to determine differences in total GRP serum concentrations between age subgroups. (b) Mann-Whitney U-Test to determine differences in tGRP serum concentrations between sexes (female *vs.* male) in each age group. tGRP - total Gla-rich protein. F - female. M - male.

From an initial recruitment of 363 volunteer blood donors, 254 participants met all eligibility criteria and were included in the final analyses. Exclusions were due to high-sensitivity C-reactive protein (hsCRP) concentrations > 3 mg/L (N = 49), estimated glomerular filtration rate < 90 mL/min/1.73 m^2^ (N = 2), total cholesterol > 5.7mmol/L (N = 27), LDL-cholesterol > 3.4 mmol/L (N = 11), and body mass index ≥ 30 kg/m^2^ (N = 3). The final study population included 121 men (47.6%). Median age was 41 (18-65) years. Descriptive demographic and biochemical characteristics of the study population are summarized in Table 1. The median serum tGRP concentration in the overall population was 1809.5 pg/mL (IQR: 1197.4-2476.0 pg/mL), Table 3. When participants were stratified into age tertiles (18-30, 31-46, and 47-65 years), serum tGRP concentrations differed across age groups, (Figure 1a; Kruskal-Wallis test, P = 0.038). Median tGRP concentrations were 2069.5 pg/mL (IQR: 1509.5-2816.0 pg/mL for 18-30 years, 1783.0 pg/mL (IQR: 1273.4-2435.6 pg/mL for 31-46 years, and 1516.0 pg/mL (IQR: 996.3-2121.0 pg/mL for 47-65 years, Table 3. No statistically significant differences in serum tGRP concentrations were observed between men and women in the overall population (Mann–Whitney U test, P = 0.420), nor within individual age groups (all P > 0.05) (Figure 1b). In healthy participants, serum tGRP concentrations showed a weak inverse correlation with age (Spearman r = − 0.30, P = 0.002). Although several additional associations reached statistical significance with tGRP, such as hsCRP (r = - 0.20, P = 0.004) and LDL-cholesterol (r = - 0.16, P = 0.003), and weakly positively correlated with estimated glomerular filtration rate (eGFR; r = 0.11, P = 0.025), serum creatinine (r = 0.14, P = 0.028), calcium (r = 0.16, P = 0.011), and phosphate (r = 0.14, P = 0.027, correlation coefficients were < 0.25 and therefore not considered meaningful. No statistically significant correlations were observed between tGRP concentrations and BMI, total cholesterol, HDL-cholesterol, total protein, magnesium, sodium, potassium, total bilirubin, or AST. When tGRP concentrations were dichotomized using the population median, differences in distribution across age groups were observed (χ^2^ test, P = 0.013; Table 4). *Post hoc* analysis using the z-test for comparison of proportions with Bonferroni correction showed that the proportion of individuals with higher tGRP concentrations was significantly greater in the youngest age group compared with the oldest age group, while no significant differences were observed between the intermediate group and the other age groups. Variables showing significant univariable associations with serum tGRP concentrations were further evaluated using stepwise multivariable linear regression. In the final model, age and serum calcium concentration remained independently associated with tGRP concentrations. Age showed an inverse association with tGRP concentrations, whereas serum calcium was positively associated. The final model explained approximately 7% of the variability in serum total GRP concentrations (Table 5).

**Table 3:** Serum total Gla-rich protein concentrations according to age groups and sex in healthy adults.

**Group**	**N**	**Median (pg/mL)**	**IQR (pg/mL)**
**Overall population**			
Total	254	1809.5	1197.4-2476.0
Male	121	1799.5	1185.3-2616.1
Female	133	1761.0	1227.0-2265.3
**Age 18-30 years**			
Total	71	2069.5	1509.5-2816.0
Male	24	2263.6	1594.8-3077.0
Female	47	2004.5	1479.5-2624.3
**Age 31-46 years**			
Total	96	1783.0	1273.4-2435.6
Male	52	1799.5	1205.8-2652.0
Female	44	1644.3	1278.4-2229.5
**Age 47-65 years**			
Total	87	1516.0	996.3-2121.0
Male	45	1571.0	1012.9-2226.0
Female	42	1506.2	948.8-2100.7
IQR - interquartile range.			

**Table 4:** Comparison of age subgroups between lower and higher total Gla-rich protein serum concentrations

		**Age subgroups (years)**			**P**	**Total individuals (N)**
		**18-30** **N (%)**	**31-46** **N (%)**	**47-65** **N (%)**		
Median for tGRP serum concentrations (pg/mL)	≤ 1809.5	27 (20.8%)^a^	50 (38.5%)^a,b^	53 (40.8%)^b^	< 0.001	130
	> 1809.5	44 (35.8%)^a^	46 (37.4%)^a,b^	33 (26.8.%)^b^		123
Total Individuals (N*)*		71	96	86		254
Data are presented as frequency of the observation found between higher tGRP concentrations (> 1809.5 pg/mL) and lower tGRP concentrations (≤ 1809.5 pg/mL) along the 3 age subgroups. Chi-squared test for differences between groups are given. *Post hoc* comparisons between age subgroups were performed using the z-test for comparison of proportions with Bonferroni correction. ^a,b^indicate statistically significant differences between groups. tGRP - total Gla-rich protein.						

**Table 5:** Factors associated with serum total Gla-rich protein concentrations

**Variable**	**B (unstandardized)**	**95% CI for B**	**β (standardized)**
Age (years)	- 18.3	- 27.5 to - 9.1	- 0.238
Serum calcium (mmol/L)	937.5	102.2 to 1773.1	0.134
Model statistics: Adjusted R^2^ = 0.073. F-change = 4.886. P = 0.028. N = 254. Multivariable linear regression analysis of factors associated with serum total Gla-rich protein (tGRP) concentrations in healthy adults. Age and serum calcium concentration were retained in the final stepwise linear regression model. Unstandardized (B) and standardized (β) regression coefficients with 95% confidence intervals and P values are shown. CI - confidence interval.			

## Discussion

In this cross-sectional study, we characterized circulating tGRP serum concentrations in a cohort of healthy adult blood donors and explored their associations with demographic and selected laboratory parameters. By focusing on a well-defined population and samples collected and stored under comparable conditions, this study provides consistent descriptive data on serum tGRP concentrations in healthy adults. These data contribute to the interpretation of GRP measurements in future population-based and clinical studies. The most consistent finding was the inverse association between serum tGRP concentrations and age. Serum tGRP concentrations differed significantly across age tertiles, with higher concentrations observed in younger adults, whereas no significant differences were detected between sexes. *Post hoc* comparisons further showed that this difference was primarily driven by higher tGRP concentrations in the youngest age group compared with the oldest group, while the intermediate group showed no significant differences. These findings indicate that age is an important factor to consider when interpreting circulating tGRP concentrations in healthy individuals.

Age-related changes have been described for other vitamin K-dependent such as Matrix Gla Protein, supporting the biological plausibility of this observation (*19*, *20*). Nevertheless, the cross-sectional design of the study does not allow conclusions regarding causality, and longitudinal studies will be required to determine whether changes in tGRP concentrations reflect aging-related physiological processes or other age-associated factors.

In univariable analyses, serum tGRP concentrations were associated with several laboratory parameters. Although some associations reached statistical significance, their correlation coefficients were low and therefore not considered clinically meaningful. In the final stepwise regression model, age and serum calcium concentration remained independently associated with serum tGRP concentrations, jointly explaining a modest proportion of their variability. This finding suggests that circulating tGRP concentrations in healthy adults are influenced by multiple determinants, and that a substantial proportion of interindividual variability remains unexplained.

The independent association between serum calcium concentration and circulating tGRP concentrations is consistent with the known biochemical properties of GRP. Gla-rich protein is a vitamin K–dependent protein with a high density of γ-carboxylated glutamate residues, which confer calcium- and mineral-binding capacity (*1*). Experimental and clinical studies have demonstrated that GRP can interact with calcium-containing mineral phases and is present in calcified tissues and extracellular matrix structures (*9*, *11*, *14*, *21*). Although the present study does not allow mechanistic inferences, the observed association between serum calcium and tGRP concentrations in healthy individuals is biologically plausible and supports the concept that circulating tGRP may be linked to systemic mineral homeostasis, even within physiological ranges (*17*). Previous studies have reported circulating GRP concentrations in control groups included in cardiovascular disease-focused studies (*15*, *16*). However, these control populations were not specifically selected to represent healthy adults and were characterized by moderate-to-high cardiovascular risk. Although similar analytical approaches were used, the reported total GRP concentration ranges differ substantially between these studies. In addition, limited clarity in the description of the biological sample matrix, with inconsistent reporting of serum *versus* plasma, further limits direct comparison and interpretation of absolute GRP concentrations (*15*). Accordingly, the present study provides, for the first time, consistent descriptive data generated using a well-characterized sandwich ELISA applied to a characterized healthy cohort. This approach enables a more robust interpretation of circulating tGRP concentrations and supports the future development of evidence-based reference frameworks, as well as clinical and epidemiological studies involving this biomarker.

The analytical validation and long-term stability assessment reported here provide methodological support for the population-based findings and strengthen confidence in the reported serum tGRP concentrations. This study has several limitations that should be acknowledged. First, participants were recruited among voluntary blood donors, and blood collection was performed without standardized fasting conditions or a fixed time window, which may have introduced additional biological variability. Second, the study population consisted exclusively of Portuguese adults of European ancestry, limiting the generalizability of the findings to other ethnic or geographic populations. Third, although age and serum calcium concentration were independently associated with serum tGRP concentrations, the explained variability was relatively modest, indicating that other factors, such as dietary habits, vitamin K status, genetic variability, or unmeasured preanalytical variables, may contribute to circulating tGRP concentrations. Finally, the cross-sectional design precludes assessment of intra-individual temporal changes.

In conclusion, this study provides robust data on circulating total GRP serum concentrations in healthy adults and demonstrates an age-associated pattern that should be considered in the interpretation of GRP measurements. The findings highlight the importance of well-characterized healthy populations and analytically validated assays in biomarker research. Future studies in larger and more diverse populations, ideally using longitudinal designs, are warranted to further elucidate the determinants and clinical relevance of circulating GRP concentrations.

## Data Availability

The data that support the findings of this study are not openly available due to reasons of sensitivity, privacy restrictions and because they are in the process of analysis for results publication, but are available from the corresponding author upon reasonable request.
